# Using WhatsApp support groups to promote responsive caregiving, caregiver mental health and child development in the COVID-19 era: A randomised controlled trial of a fully digital parenting intervention

**DOI:** 10.1177/20552076231203893

**Published:** 2023-11-03

**Authors:** Sarah Skeen, Marguerite Marlow, Stefani du Toit, GJ Melendez-Torres, Lynette Mudekunye, Edwick Mapalala, Kelvin Ngoma, Byamukama Michael Ntanda, Moroesi Maketha, Caitlin Grieve, Laura Hartmann, Sarah Gordon, Mark Tomlinson

**Affiliations:** 1 121470 Institute for Life Course Health Research, Stellenbosch University, Tygerberg, South Africa; 2 100441 Amsterdam Institute for Social Science Research, University of Amsterdam, Amsterdam, Netherlands; 3 Department of Psychiatry and Mental Health, 37716 University of Cape Town, Rondebosch, South Africa; 4 Peninsula Technology Assessment Group (PenTAG), 3286 University of Exeter, Exeter, UK; 5 194164 Regional Psychosocial Support Initiative (REPSSI) Regional, Randburg, South Africa; 6 Regional Psychosocial Support Initiative (REPSSI), Dar es Salaam, Tanzania; 7 Regional Psychosocial Support Initiative (REPSSI), Lusaka, Zambia; 8 Regional Psychosocial Support Initiative (REPSSI), Kampala, Uganda; 9 121470 Department of Global Health, Centre for Evidence-Based Health Care, Stellenbosch University, Tygerberg, South Africa; 10 School of Nursing & Midwifery, Queen's University Belfast, Belfast, Northern Ireland

**Keywords:** Digital, parenting, child development, responsive caregiving, mental health, COVID-19

## Abstract

**Objective:**

Digital interventions hold important potential for supporting parents when face-to-face interventions are unavailable. We assessed the feasibility and effectiveness of a digital parenting intervention in Zambia and Tanzania.

**Methods:**

Using a randomised controlled trial, we evaluated the Sharing Stories digital parenting intervention for caregivers of children aged 9–32 months with access to a smartphone in their household. Caregivers were stratified based on child age and randomly assigned to the intervention or waitlist control arm. The intervention was delivered via facilitated WhatsApp groups over 6 weeks to promote caregiver wellbeing and responsive caregiving through shared reading activities. Primary outcomes were caregiver-reported responsive caregiving, child language and socio-emotional development. Secondary outcomes were caregiver mental health and parental stress. Masked assessors conducted assessments at baseline and immediate follow-up.

**Results:**

Between October 2020 and March 2021, we randomly assigned 494 caregiver–child dyads to the intervention (*n* = 248) or waitlist control (*n* = 246) arm. Caregivers in the intervention group reported more responsive caregiving (OR = 2.55, 95% CI: 1.15–5.66, *p *= 0.02), time reading or looking at books (β = 0.45, *p *= 0.04) and telling stories (β = 0.72, *p *= 0.002). Intervention caregivers reported significantly lower symptoms of depression (β = −0.64, *p *= 0.05) and anxiety (β = −0.65, *p *= 0.02). Child development and parental stress did not differ significantly between groups.

**Conclusions:**

Digital parenting interventions using WhatsApp can effectively promote responsive caregiving and caregiver mental health in low-resource settings, with great potential for scalability.

**Trial registration:**

ISRCTN database, ISRCTN77689525.

## Introduction

Young children require nurturing, emotionally supportive environments to help them grow and develop optimally. Parents and caregivers can benefit from additional support in their roles, particularly in settings of adversity where caregiving capacity may be under strain.^1,2^ There is a substantial evidence base that clearly demonstrates that universal parenting support interventions delivered during the first 3 years of life are effective in improving both caregiving behaviours and child development outcomes, with pronounced benefits for caregivers in low- and middle-income country (LMIC) settings.^3^ Scale-up of these in-person evidence-based interventions can be hampered by availability of human resources and logistical issues.^4^ There has been a significant increase in programmes using digital or technology-assisted delivery methods for evidence-based interventions as a means to support scale-up, notably in the field of mental health,^5^ maternal and neonatal health^6^ and child survival and development.^7^ Similarly, for parenting support, there is evidence that digitally delivered or technology-assisted interventions can overcome some of these challenges.^8,9^ Increasingly, social media platforms provide opportunities for reaching and connecting groups of caregivers to address parenting and child wellbeing.^7^

The COVID-19 pandemic substantially increased stressors faced by caregivers and children, while simultaneously disrupting their access to services and support. As a result, the pandemic had devastating effects on children and families globally, and especially in resource-deprived settings, which will persist into the future.^10^ Yet, the onset of the pandemic also provided impetus for the scale-up of digital solutions to improve service access and necessitated new models of reaching and supporting caregivers.^11^ As a result, a number of global-, regional- and national-level agencies released practical, evidence-based, online resources, intended to improve parents’ knowledge and provide ideas for stimulating activities. An evaluation of this effort showed that the campaign provided timely information for caregivers that they would not have been able to access during COVID-19 restrictions.^12^ There is an on-going need for digitally delivered programmes that both provide information and develop and strengthen skills to foster nurturing relationships in an engaging and supportive environment. However, while remote-based methods are increasingly perceived as feasible,^13,14^ digital interventions can be inaccessible to caregivers in low-resource settings due to issues with connectivity, access to digital devices, cost, digital and functional literacy and availability of content in local languages.^15^

There is limited evidence on the effectiveness of digitally delivered parenting interventions from LMICs to improve caregiving behaviours, caregiver mental health and child outcomes. Our study is one of the first to look at the feasibility, acceptability and effectiveness of using WhatsApp groups to offer both parenting and mental health support to caregivers of young children in two LMICs in the African region. We adapted existing evidence-based in-person interventions to improve child development, responsive caregiving and caregiver mental health support to create the digitally delivered ‘Sharing Stories’ intervention for use in low-resource contexts.

## Methods

### Study design and participants

The Sharing Stories Project was a waitlist randomised controlled trial conducted in Tanzania and Zambia. The site of the study in Tanzania was urban, with participants recruited from southern and northwest areas of Dar es Salaam. The site in Zambia was mainly rural, with participants recruited from three rural farming districts and one urban district in central Zambia. Tanzania has 85 mobile phone subscriptions per 100 people, with 95% mobile network coverage, while Zambia has 104 mobile phone subscriptions per 100 people and 78% mobile network coverage.^16^ Initially the trial was also due to take place in Uganda. Enrolled participants in Uganda completed baseline assessments, but due to several unforeseen delays, relating to the 2021 national election and a prolonged nationwide shutdown of social media platforms (the delivery platform of the intervention), we were not able to deliver the programme. The trial was suspended, and no follow-up data were collected.

The trial was registered on the International Standard Randomized Controlled Trial Number database (ISRCTN77689525).

Eligible participants were adult primary caregivers of children between 9 and 32 months of age at the time of recruitment. Adult primary caregivers were eligible if they were 18 years or older, had access to a working smartphone in their household and consented to participate. Participants were recruited through local community-based organisations and their networks in each study site. Using existing programme databases, project staff contacted caregivers via an initial phone call or a WhatsApp message to inform them of the study and invite them to participate. Recruiters followed a script with a set of questions to assess eligibility, with responses captured on data collection software on a mobile device. In addition, staff asked participants to identify other caregivers within their own social networks who might be interested in participating in the study. All interested participants were assessed for eligibility before being formally recruited into the study. All caregivers provided written informed consent at the time of baseline data collection before randomisation.

### Randomisation and masking

Caregiver–child dyads were the unit of randomization. Caregivers were stratified based on child age (9–20 months or 21–32 months) and randomly assigned to either the intervention or waitlist control arm after baseline assessments. Randomisation was completed by an independent statistician using a web-based randomisation programme. All data collectors were masked to group allocation in order to minimise assessment bias. Due to the nature of the intervention, masking of participants and intervention facilitators who delivered the intervention was not possible. All efforts were made to keep data collection teams masked to group status. This included training study staff on the importance of reducing bias in trials and by ensuring that intervention and data collection teams worked independently and did not share data. All caregivers were instructed at the start of the follow-up interview to refrain from mentioning to data collectors programme activities that they may or may not have received. Caregivers in the waitlist control arm did not receive the waitlist intervention until after the follow-up data were collected, at which point they completed the programme.

### Sample size

The sample size was based on the calculations done for a previous evaluation of an in-person version of the programme conducted in South Africa.^17^ This previous trial used a sample of 140 caregiver–child dyads (70 in each arm), based on a calculation at 80% power and *α* = 0.05 and including 10% dropout, to detect moderate differences in child language development and social and emotional development. Based on this, in the current study, we oversampled by 70% in each site given the significant uncertainty about digital programme uptake and rates of dropout in the context of COVID-19.

### Intervention

The Sharing Stories intervention consists of two primary components. The first is focused on using shared reading activities to encourage responsive caregiving and promote child development. Caregivers receive digital books via their phones and guidance on how to use them to engage their children in playful and responsive ways. The second component addresses caregiver mental health by providing caregivers with information and support in changing negative thought patterns, dealing with difficult emotions, and strategies for accessing support. The programme is based on two WHO-endorsed evidence-based interventions, the Parenting for Lifelong Health shared reading intervention and the Thinking Healthy programme, both of which have been used and evaluated extensively in LMIC settings.^17–20^ To maximise the likelihood of programme success, consultations were conducted with 15 caregivers and 15 programme implementers across the study countries to inform the adaption of the content and to ensure that contextual preferences and priorities were incorporated into the programme.

The intervention was delivered fully remotely to groups of 30–40 caregivers concurrently via facilitated WhatsApp groups over a 6-week period. Each week, facilitators hosted a group chat session (lasting 1–2 h), where new content was introduced (see Table 1), with opportunity for questions and discussion via chat. Content was presented using text, voice notes, infographics (images combined with minimal text), with animations and video clips of caregivers and children to model key skills. Between weekly group chat sessions, facilitators sent three different recap messages for continued engagement with caregivers. Caregivers received two new digital picture books each week and an additional four books once the programme had ended (16 digital books in total). All intervention content was translated and presented in the local languages.

**Table 1.** Responsive caregiving and mental health content focus areas per session.

Session	Responsive caregiving/shared reading	Caregiver wellbeing
Session 1	Creating positive experiences The importance of quality time; using books and stories to have fun together; having shared experiences; using a lively, playful voice to engage your child.	Taking care of yourself Acknowledging the demands of caregiving; prioritising your own wellbeing (caring for others starts with caring for yourself); making time for self-care; self-care activities.
Session 2	Following your child's lead Taking notice of what your child is interested in; follow your child's lead; don’t force their participation; respond in a positive way; use praise and encouragement.	Be kind to yourself Being aware of your thoughts and feelings; being kind and gentle towards yourself; think of what you do well; think about what you are grateful for.
Session 3	Pointing and naming Using ‘pointing and naming’ to help your child learn new words and how to use them. Using positive responses to encourage their learning.	Taking control of your thoughts Acknowledging how stress can affect your thoughts; changing the way you think; taking control of your thoughts; turning unhelpful thoughts into helpful thoughts.
Session 4	Mimicking actions and making links Bring the pictures to life; mimic sounds and actions from the story; make links between the pictures and your child's world.	Asking for help and support Asking for help; talking to someone you trust; surrounding yourself with people who support and value you.
Session 5	Talking about feelings Talking to your child about different emotions (your child's feelings and the feelings you see in the pictures); making links between the feelings in the pictures and your child's feelings; supporting your child's feelings.	Coping with stress Using positive coping mechanisms to overcome unwanted emotions (focus on healthy activities; be kind to yourself; taking breaks; breathing techniques).
Session 6	Encouraging your child to be curious Encouraging your child to talk and ask questions; using ‘who, what, where, why, how’ questions; asking questions that help your child think about what is happening and why.	Reflection Reflecting on what you have learnt and what has changed; recap of the previous 5 weeks’ content.

To create the WhatsApp groups, caregivers were ranked according to child age (youngest to oldest) and divided into three groups so that caregivers with children of a similar age would be in the same WhatsApp groups. Caregivers were added to their group a week prior to the start of the programme to confirm that their contact details were correct. Each enrolled caregiver received a data bundle to enable them to engage in the groups and access the content. Facilitators used a manual with step-by-step instructions for posting content in the groups. They monitored group discussions to ensure that all engagement was respectful and remained on-topic and to assist with technical difficulties. Between contact points, posting by group members was restricted.

Two trained lay community facilitators worked together to present content to each group, moderate group discussions and support caregivers to implement and practise their skills. Facilitators received remote training over a 5-day period, using Zoom, focused on responsive caregiving and caregiver wellbeing and shared reading techniques and group facilitation. Training was designed to be interactive, using video demonstration, discussion and practice. In addition, facilitators were trained to create WhatsApp groups, manage settings and post different forms of content (e.g. video and audio files) into the groups. Practice groups were run before the start of the intervention to allow facilitators to become familiar with the implementation procedures. For the duration of the programme, facilitators were supported through a WhatsApp supervision chat group, in addition to weekly supervision meetings. The WhatsApp group enabled facilitators to request support in real time (for example, for assistance with responding to a caregiver's question or with any issues in posting content). Supervisors reviewed the content posted on the groups each week to ensure fidelity to the manual. The weekly supervision meetings were used for debriefing following the week's sessions and reviewing content for the following week.

### Outcomes

Primary outcomes were responsive caregiving behaviours, child language development and child social and emotional development. Secondary outcomes were caregiver mental health (depression and anxiety symptoms) and parental stress.

We measured responsive caregiving using the Parent–Child Conflict Tactics Scale^21^ (PCTS) and selected items from the (i) responsiveness and acceptance and (ii) support for learning subscales of the family care indicators (FCI).^22^ Child language was assessed using the Caregiver Reported Early Development Instrument (CREDI) language long form.^23^ We measured child social and emotional development using the CREDI social–emotional long form, the Strengths and Difficulties Questionnaire^24^ (SDQ) and the aggression subscale of the Child Behavior Checklist (CBCL).^25^ For the CREDI, higher scores indicate better child development outcomes, while for the SDQ and CBCL, higher scores indicate more problematic child behaviour.

We measured caregiver mental health using the Patient Health Questionnaire-9^26^ (PHQ-9) for depression symptoms and the Generalized Anxiety Disorder-7^27^ (GAD-7) for anxiety symptoms. Parental stress was assessed using the Parental Stress Scale (PSS) short form.^28^ For the PHQ-9, GAD-7 and PSS, higher scores indicate worse mental health outcomes.

Outcome data were collected at baseline and directly post-intervention, with process data collected during programme implementation. Prior to data collection, all research staff received training on the study procedures, with refresher training workshops conducted between data collection time-points. Following informed consent, caregivers participated in a telephonic interview with a trained data collector, who captured participant responses on a mobile device. All interviews were conducted in the local language. As a measure of quality assurance, interviews were audio-recorded and checked to ensure accurate data capturing.

### Statistical analyses

We considered outcomes in three categories based on their measurement approach and used distinct analysis approaches for each. All analyses were undertaken by country before pooling the data with country added as an additional covariate. We undertook all statistical analyses in Stata v 16.

The first category comprised the CREDI language and social–emotional subscales. Individuals were scored at baseline and follow-up using published algorithms,^23^ generating for each child and scale, a raw score (as opposed to a z-score) and a standard error of measurement. Analysis was via linear regression including terms for group allocation and stratification factor, with observations weighted using the inverse variance survey weights derived from the standard errors of measurement. Non-normality of residuals was addressed via robust standard errors. Because a significant number of children ‘aged through’ different CREDI items throughout the trial, we did not control for baseline values nor attempt imputation of missing data for this outcome category.

The second category comprised the SDQ and CBCL. Analysis was via linear regression including terms for group allocation and stratification factor. Non-normality of residuals was addressed via robust standard errors. Because not all SDQ subscales were collected at baseline and because children could ‘age through’ these assessments, we did not control for baseline values nor attempt imputation of missing data.

The third category comprised positive, responsive parenting behaviours and all secondary outcomes. As there was no aspect of these measures related to child age, multiple imputation could be used to address missingness. Multiple imputation was undertaken using fully conditional specifications. Continuous variables were imputed using predictive mean matching with five nearest neighbours to account for non-normality of residuals. Binary variables were imputed using a logit link with augmentation to avoid complete separation. All imputations were stratified by a four-category variable defined by crossing country with group allocation. Twenty imputations were undertaken. Analysis for continuous variables was via linear regression including terms for group allocation, stratification factor and baseline value, with robust standard errors. Analysis for binary outcomes was via logistic regression with terms for group allocation and stratification factor.

### Role of the funding source

The study was funded by the LEGO Foundation. The funders had no role in the detail of study design, data collection, data analysis, data interpretation or writing of the report.

## Results

**Figure 1.**
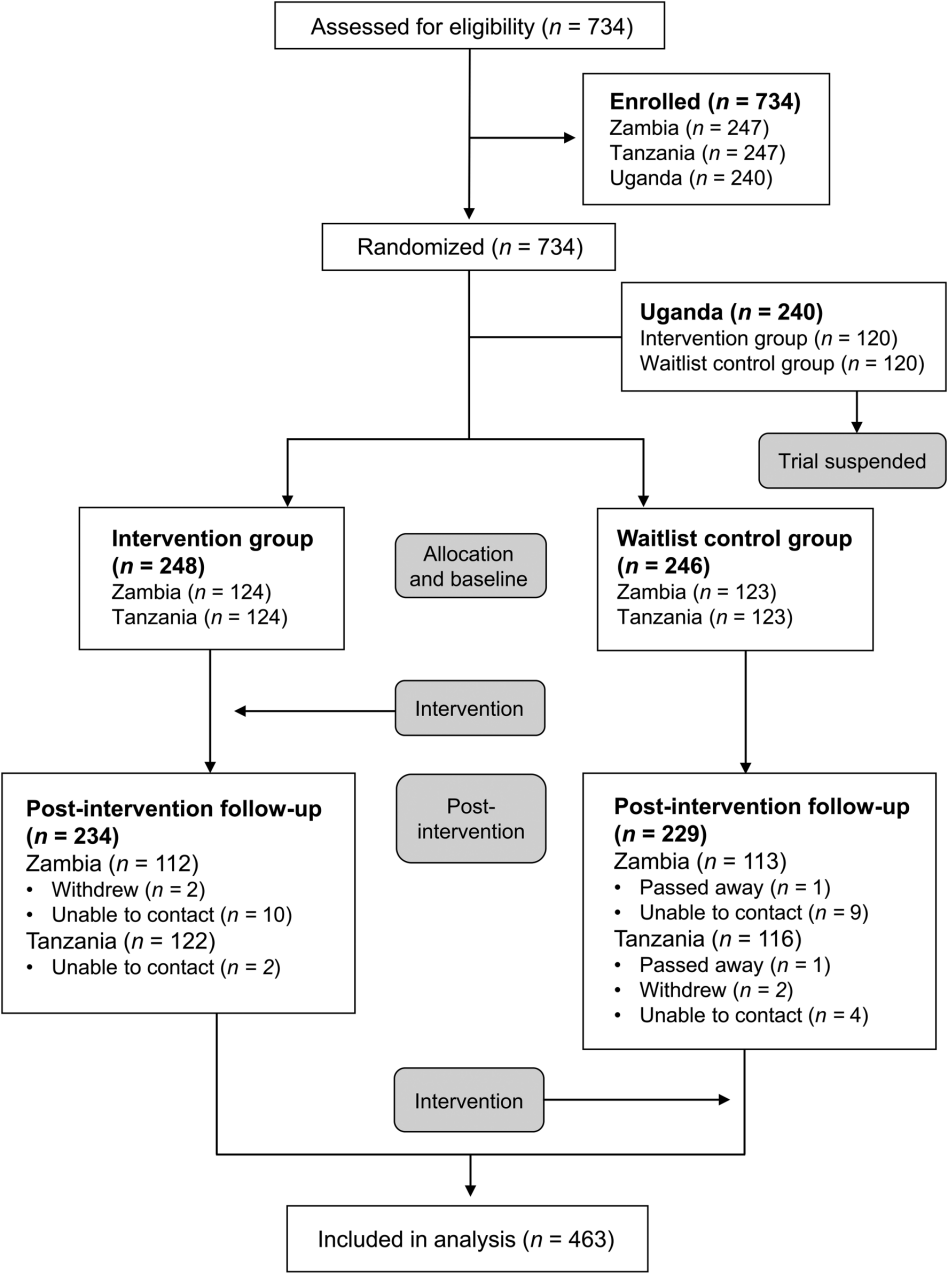
CONSORT diagram.

Between October 2020 and March 2021, 494 caregiver–child dyads were enrolled and randomly assigned to either the digital parenting intervention (*n* = 248) or the waitlist control (*n* = 246), with a post-intervention follow-up assessment rate of 94% (Figure 1).

Both countries had a total of 247 caregivers (124 caregivers in the intervention arm and 123 in the control arm). At baseline, there were no statistically significant differences amongst groups in any of the child characteristics (age and gender) or caregiver characteristics (age, gender, depression symptoms, anxiety symptoms, marital status, education and employment) (Table 2).

**Table 2.** Baseline sociodemographic characteristics.

	Pooled sample (*n* = 494)		Zambia (*n* = 274)		Tanzania (*n* = 274)	
	Control arm (*n* = 246)	Intervention arm (*n* = 248)	Control arm (*n* = 123)	Intervention arm (*n* = 124)	Control arm (*n* = 123)	Intervention arm (*n* = 124)
Child						
Age, months	20.10 (7.17)	20.56 (7.17)	19.9 (7.1)	20.0 (7.0)	20.3 (7.2)	21.1 (7.3)
Gender						
Male	111 (45%)	114 (46%)	57 (46%)	64 (52%)	54 (44%)	50 (40%)
Female	135 (55%)	134 (54%)	66 (54%)	60 (48%)	69 (56%)	74 (60%)
Caregiver						
Age, years^A^	31.39 (7.97)	31.59 (8.08)	30.9 (9.2)	31.3 (9.2)	31.9 (6.7)	31.9 (6.8)
Gender						
Female	190 (77%)	188 (76%)	96 (78%)	90 (73%)	94 (76%)	98 (79%
Male	56 (23%)	60 (24%)	27 (22%)	34 (27%)	29 (24%)	26 (21%)
Depression (PHQ-9)^A^	4.88 (4.36)	5.10 (4.15)	5.80 (4.55)	6.39 (4.15)	3.97 (3.98)	3.82 (3.75)
Anxiety (GAD-7)^A^	3.81 (3.50)	4.29 (4.05)	4.55 (3.60)	5.37 (4.41)	3.07 (3.25)	3.21 (3.32)
Marital status						
Married / Living with partner	166 (67%)	176 (71%)	73 (59%)	78 (63%)	93 (76%)	98 (79%)
Single	80 (33%)	72 (29%)	50 (41%)	46 (37%)	30 (24%)	26 (21%)
Education						
No schooling / some primary schooling	50 (20%)	47 (19%)	13 (11%)	11 (9%)	37 (30%)	36 (29%)
Some secondary schooling	127 (52%)	123 (50%)	80 (65%)	82 (66%)	47 (38%)	41 (33%)
Tertiary qualification	69 (28%)	78 (31%)	30 (24%)	31 (25%)	39 (32%	47 (38%)
Employment						
Unemployed	100 (41%)	100 (40%)	70 (57%)	66 (53%)	30 (24%)	34 (27%)
Full time	26 (11%)	21 (9%)	22 (18%)	16 (13%)	4 (3%)	5 (4%)
Part time / self employed	120 (48%)	127 (51%)	31 (25%)	42 (34%)	89 (72%)	85 (69%)

^A^ Mean and standard deviation are reported.

### Primary outcomes

Post-intervention, for the pooled sample, caregivers in the intervention group were significantly more likely to be engaging in responsive caregiving practices (OR = 2.55, 95% CI: 1.15–5.66, *p *= 0.02) than caregivers in the control group. Caregivers in the intervention group were also significantly more likely to spend time reading or looking at picture books with their child (β = 0.45, *p *= 0.04) and/telling their child stories (β = 0.72, *p *= 0.002) than caregivers in the control group. No significant improvements in child social and emotional development or child language were observed in the intervention group when compared to children in the control group (Table 3).

**Table 3.** Effects of intervention on responsive caregiving, child language and social and emotional development.

	Pooled sample (*n* = 494)				Zambia (*n* = 247)				Tanzania (*n* = 247)			
	Coef.	*p*-value	95%	CI	Coef.	*p*-value	95%	CI	Coef.	*p*-value	95%	CI
Primary outcomes												
Positive, responsive parenting behaviours												
Parent–Child Conflict Tactics (PCTS)	−0.10	0.70	−0.63	0.43	−0.15	0.74	−1.02	0.73	−0.06	0.85	−0.68	0.55
Beliefs about physical punishments (PCTS)^a^	1.04	0.87	0.67	1.61	1.25	0.42	0.72	2.17	0.73	0.41	0.34	1.55
Caregiver responsiveness (FCI)^a^	2.55	0.02*	1.15	5.66	1.45	0.51	0.48	4.45	4.49	0.02*	1.32	15.28
FCI 2 (hours a day child watch TV?)	0.01	0.88	−0.15	0.18	−0.04	0.76	−0.30	0.22	0.06	0.58	−0.15	0.27
FCI 3 (read books or look at picture books with child)	0.45	0.04*	0.02	0.90	0.41	0.23	−0.26	1.08	0.54	0.08	−0.06	1.13
FCI 4 (tell the child stories)	0.72	0.002**	0.28	1.17	0.65	0.06	−0.03	1.32	0.80	0.01**	0.17	1.42
FCI 5 (sing songs to child)	0.16	0.50	−0.30	0.62	0.23	0.48	−0.41	0.87	0.08	0.80	−0.57	0.74
FCI 7 (play with the child)	−0.17	0.39	−0.57	0.23	−0.24	0.31	−0.70	0.22	−0.08	0.80	−0.71	0.55
FCI 8 (spend time in learning activities with the child)	0.05	0.83	−0.42	0.52	0.26	0.44	−0.41	0.93	−0.14	0.68	−0.81	0.53
FCI 13 (talk to child during meals)	0.46	0.07	−0.03	0.94	0.65	0.08	−0.07	1.37	0.27	0.43	−0.41	0.95
Child language development												
Language development (CREDI)	0.18	0.06	−0.01	0.36	0.17	0.20	−0.09	0.43	0.17	0.19	−0.09	0.45
Child social and emotional development												
Socio-emotional development (CREDI)	0.12	0.21	−0.07	0.32	0.06	0.65	−0.20	0.32	0.17	0.23	−0.11	0.46
Prosocial behaviour (SDQ)	−0.42	0.09	−0.92	0.06	−0.34	0.33	−1.04	0.36	−0.51	0.16	−1.20	0.20
Hyperactivity–inattention (SDQ)	0.17	0.42	−0.25	0.60	0.16	0.63	−0.51	0.83	0.19	0.50	−0.36	0.74
Emotional symptoms (SDQ)	0.02	0.92	−0.33	0.37	−0.04	0.87	−0.55	0.47	0.07	0.77	−0.41	0.55
Conduct problems (SDQ)	−0.03	0.89	−0.45	0.39	−0.21	0.50	−0.81	0.40	0.13	0.67	−0.47	0.73
Peer problems (SDQ)	0.27	0.08	−0.04	0.59	0.08	0.73	−0.36	0.52	0.46	0.04*	0.01	0.91
SDQ total difficulties	0.44	0.38	−0.57	1.46	−0.01	0.99	−1.35	1.34	0.84	0.27	−0.66	2.35
Aggressive behaviour (CBCL)	0.51	0.34	−0.55	1.59	0.20	0.79	−1.31	1.72	0.82	0.29	−0.70	2.39

^a^ Odds ratio reported.

*p ≤ 0.05; ** *p* ≤ 0.01.

CREDI: Caregiver Reported Early Development Instrument; SDQ: Strength and Difficulties Questionnaire; CBCL: Child Behaviour Checklist; PCTS: Parent–Child Conflict Tactics scales; FCI: Family Care Index.

For Zambia, no child development or parenting outcomes were statistically significant; however, a positive trend (*p* < 0.1) was detected for telling the child stories (β = 0.65, 95% CI: −0.03–1.32, *p* = 0.06) and talking to the child during meals (β = 0.65, 95% CI: −0.07–1.37, *p* = 0.08). For Tanzania, caregivers in the intervention group were significantly more likely to engage in responsive caregiving practices (OR = 4.49, 95% CI: 1.32–15.28, *p* = 0.02) and spending time telling their child stories (β = 0.80, *p* = 0.01). Caregivers reported significant increases in observed peer problems in intervention group children compared to controls (β = 0.46, *p *= 0.04).

### Secondary outcomes

For the pooled sample, caregivers in the intervention group reported significantly lower levels of depression (β = −0.64, *p *= 0.05) and anxiety (β = −0.65, *p *= 0.02) symptoms at follow-up compared to caregivers in the control group. No significant group differences in parenting stress (β = −0.67, *p *= 0.33) were observed. In Zambia, significant lower levels of depression (β = −1.83, *p *= 0.001) and anxiety (β = −1.57, *p *= 0.001) were reported. For Tanzania, none of the secondary outcomes were statistically significant (Table 4).

**Table 4.** Effects of intervention on caregiver mental health and parental stress.

	Pooled Sample (*n* = 494)				Zambia (*n* = 247)				Tanzania (*n* = 247)			
	Coef.	*p*-value	95%	CI	Coef.	*p*-value	95%	CI	Coef.	*p*-value	95%	CI
Secondary outcomes												
Caregiver mental health												
Depression (PHQ-9)	−0.64	0.05*	−1.28	−0.01	−1.83	0.001**	−2.90	−0.75	0.53	0.11	−0.12	1.18
Anxiety (GAD-7)	−0.65	0.02*	−1.17	−0.12	−1.57	0.001**	−2.45	−0.68	0.31	0.27	−0.24	0.86
Parenting stress												
Parental stress (PSS)	−0.67	0.33	−2.03	0.68	−1.60	0.08	−3.36	0.17	0.26	0.81	−1.82	2.34

*p ≤ 0.05; ** *p* ≤ 0.01.

PHQ-9; Patient Health Questionnaire-9; GAD-7; Generalized Anxiety Disorder-7; PSS; Parental Stress Scale.

During the 6-week intervention, facilitators also monitored if any caregivers in the intervention arm exited their WhatsApp group and, where possible, conducted follow-ups to establish why caregivers had decided to leave the group. Across both countries, a very small number (*n* = 5) of caregivers exited the WhatsApp groups. Reasons provided for leaving the groups included confusion about the groups opening and closing at different times and caregivers changing the contact number they wanted to participate with. Caregivers who provided these reasons requested to be added back into the groups.

## Discussion

This study demonstrated that a brief, universally delivered digital parenting intervention, delivered via WhatsApp-based support groups, improved responsive caregiving and caregiver mental health in different LMIC settings. This evaluation adds evidence to a growing body of research on the implementation and evaluation of digital group-based parenting interventions in sub-Saharan Africa. The unique aspect of the intervention is that it is delivered entirely remotely and combines a focus on caregiver emotional wellbeing with strengthening positive, responsive caregiving while simultaneously providing skills for child stimulation through the use of digital picture books.

Across the full sample, the intervention improved responsive caregiving practices, with caregivers reporting being more responsive to their children's needs, spending more time with their children reading or looking at picture books and telling their child stories. These aspects of responsive caregiving and creating opportunities for early learning are central to the type of nurturing care that supports children's learning and development across the life course.^2^ In addition, for the pooled sample, the intervention successfully lowered caregivers’ symptoms of depression and anxiety. Caregiver mental health is a critical resource for responsive, playful caregiving; however, few interventions focus on the emotional wellbeing of caregivers as part of ECD programmes.^3^

The intervention did not significantly improve child development outcomes. This is in contrast with three previous evaluations of the in-person version of the intervention, each of which demonstrated improvements in child development, particularly child language.^17–19^ Two important differences between the digital and in-person interventions should be noted. First, the in-person intervention is usually delivered over eight sessions, instead of six, and focused only on responsive caregiving, not caregiver mental health. It is therefore possible that improving child language and socio-emotional development requires longer and more focused intervention and that brief, digital group-based interventions are simply not sufficient to produce improvements in these domains. Second, the in-person version allows for brief one-on-one practice opportunities between caregivers and their children while the facilitator offers feedback and support. This aligns with research showing that caregiver education alone is ineffective in improving child outcomes and didactically focused parent groups are less effective than those that include role-play or practice opportunities.^29^ Future iterations of the digital version of the programme might benefit from more specific training and demonstration videos for caregivers on how to use digital books. The digital version of the intervention could also be modified to include a similar ‘practice’ component as the in-person version, for example, by inviting caregivers to send brief videos of themselves engaging in shared reading with their child and for the facilitators to comment and provide feedback to caregivers via the WhatsApp group or private chat. Alternatively, a longer follow-up period may be required to allow for the improvements in caregiving capacity and responsive caregiving to translate into observed improvements for child development outcomes. This field of work could benefit from longer-term follow-up of participants to help answer important questions regarding the duration of effects on child outcomes and potential long-term benefits of such brief, digital group-based programmes for caregivers and their children.

While the intervention demonstrated significant improvements in responsive caregiving behaviours and caregiver mental health in the pooled sample, country differences in programme impact were observed. The intervention resulted in significant improvements in parenting behaviours for caregivers in Tanzania, but not for caregivers in Zambia. In terms of mental health, the opposite was found, where the intervention significantly improved caregiver mental health outcomes in Zambia, but not in Tanzania. This could be due to differences in government responses to the pandemic. In Tanzania there was no lockdown or stringent restrictions to mitigate the spread of COVID, and schools and childcare centres remained open, potentially placing less parenting strain on caregivers when compared to Zambia. In addition, we observed higher mean scores at baseline for depression and anxiety symptoms in the rurally based sample from Zambia, meaning that the intervention may have led to greater levels of improvement for those experiencing heightened distress for this group.

### Implications for research and practice

The intervention was delivered using WhatsApp, a widely available mobile phone application in many LMICs, in local languages. The intervention was delivered in a group format by trained community facilitators, based on a manualised curriculum, with supportive supervision for facilitators. Our use of trained community facilitators as delivery agents and relatively large group sizes (30–40 caregivers per group) makes our model potentially scalable to other settings in which potential demand outpaces supply of in-person parenting support programmes.

Developers of face-to-face parent training interventions are often concerned with the degree to which an intervention is conducted according to protocol at scale.^30^ This design promotes high fidelity to the manualised programme, as facilitators deliver pre-designed, multi-media content in a structured format, with close monitoring of the groups by supervisors in real time. Bridging the gap between efficacy study and large-scale programming will depend greatly on strategies to support the management and supervision of intervention staff. The research group trained and closely supervised the programme facilitators, and, for future scale-up, these activities would have to be performed and sustained by local organisations or district government staff. One challenge will be how to ensure quality implementation at scale. A benefit of supervision of digital intervention delivery is that it allows for constant monitoring of implementation in real time and for ensuring effective supervision and accountability, in ways that are much more difficult and expensive in face-to-face programmes.

Digital delivery helps overcome some of the common logistical barriers associated with attendance of in-person parenting groups such as time to attend sessions, childcare arrangements and travel^30^ offering a more cost-effective approach. The digital delivery format offers caregivers more flexibility in terms of when they engage with the programme and its content, enabling them to catch up or revisit content in their own time. Importantly, 45% of participating caregivers were male, where previous research highlights the significant underrepresentation of fathers and male caregivers in parenting interventions.^31^ The use of media and mobile phone messaging can be advantageous to overcome some of the barriers associated with in-person attendance that disproportionately affect fathers.^32^

In addition, digital delivery facilitates opportunities for other caregivers to access messages and content. Most child development interventions target a single caregiver within a family. By storing content on phones, digital delivery of the programme creates the potential for spill-over from the participating caregiver to other household members who are also involved in caring for young children. Given the potential cost benefits of spill-over effects, exploring the potential of the programme to engage the family is essential.

We found that WhatsApp is an appropriate delivery platform, given that it is widely used, supports a range of different types of messages (text, voice, graphic and video) and is usable on relatively basic and commonly owned mobile phones, not only more expensive models. These features enable an interactive, visual and audio learning experience, along with supportive group environments that would not be possible through other delivery approaches, such as radio, or via content delivered to more basic mobile phone devices. However, while mobile phone access is increasing, considerations related to the use of phones that support WhatsApp and who has access to them, memory space on devices and data to access the intervention content will need to be prioritised if the intervention is delivered at a larger scale. It is important to consider that the factors that enable participation in the programme (access to a smartphone, a certain level of literacy, living in an area with network coverage) are not present for all families. Future efforts could explore the potential of hybrid models within communities – where families with smartphones participate in the digital version of the programme, allowing in-person resources and services to be directed at families who do not have access to a smartphone or who require more direct support. However, increasing access to smartphones for certain households might still offer a more cost-effective solution, especially where in-person delivery is not feasible. Phone sharing between households could be a potential solution, as has been previously used in women's groups to promote breastfeeding in Nigeria. Women shared a single phone and elected one group member to control the phone and share the messages.^33^

A digital delivery approach offers many benefits, especially in terms of reaching a larger number of caregivers and overcoming many logistical challenges associated with in-person delivery, therefore saving on costs. On the other hand, in-person delivery potentially reaches more vulnerable families (i.e. those who live in areas without network coverage and those who do not have access to a smartphone) and is not dependent on technology or electricity. In addition, the in-person approach allows for more opportunities to practise in the presence of a facilitator and receive individualised feedback and support. Indeed, the advantage of in-person interventions have been linked to impact through meaningful feedback and human support.^34^ As such it is vital for digital interventions to retain key elements of human connection as far as possible through approaches such as tailored feedback, opportunities for accountability and social support. A combination approach, where caregivers attend less frequent in-person sessions complemented by digital support groups, could serve to reduce in-person costs while still benefiting from the remote support approach.

The intervention was evaluated in two African countries, and future work should focus on validation across a broader set of contexts to ensure it is generalisable. The evidence base for interventions is markedly skewed towards methods and results from small, highly controlled RCTs. However, implementation science shows that effective interventions are often significantly diluted when implemented in ‘real world’ contexts and that moving from efficacy to effectiveness and eventual scale-up and dissemination is a complex undertaking. Testing different combinations of digital and in-person approaches will be important to establish which options optimise impact and minimise costs. The most compelling option would be to test a digital-only version of the programme against a version where caregivers meet once in the beginning and once at the end of the intervention, against one where caregivers meet more frequently throughout the programme. Simultaneously, solutions to improving access to the digital programme for households who do not own smartphones should be explored.

### Limitations

The study's strengths are its high rates of retention and follow-up and its evaluation of various interlinked domains of child development, responsive caregiving and caregiver mental health. However, the study also had certain limitations. First, because data collection took place telephonically, all outcomes were self-report or caregiver-report. Validated caregiver-report measures of child outcomes available for this age group for these contexts are lacking. As a result, we were only able to compare groups on certain measures, which limited the interpretation of the clinical significance of the findings. Future efforts would benefit from triangulation of data through additional direct assessment and observation – both for child development outcomes and caregiver–child interactions.

Second, the measures used in the study were not validated for telephonic use, and it is possible that the data collection process may have been influenced negatively by this approach. However, it is also possible that reporting behaviours telephonically, especially for sensitive topics such as mental health and child discipline, could have allowed caregivers to report behaviours more truthfully.

Another potential limitation to consider is the risk of contamination, as participants were members of the same social networks, invited by each other to participate in the study. It is therefore possible that caregivers in the intervention condition may have shared the intervention content with caregivers in the control condition, which could have resulted in an underestimation of the treatment effect.

## Conclusion

Digital parenting interventions using WhatsApp support groups, facilitated by trained and supervised lay community facilitators, can effectively promote responsive caregiving and improve caregiver mental health in low-resource settings, with a great potential for scalability. The economic, health and social consequences of the COVID pandemic and associated stressors are likely to impact caregivers and children for a long time. Leveraging low-cost, scalable, digital solutions such as the Sharing Stories intervention to mitigate risks for caregivers and children should form a key part of the continued response to the pandemic in the region.

## Supplemental Material

sj-docx-1-dhj-10.1177_20552076231203893 - Supplemental material for Using WhatsApp support groups to promote responsive caregiving, caregiver mental health and child development in the COVID-19 era: A randomised controlled trial of a fully digital parenting intervention Click here for additional data file. Supplemental material, sj-docx-1-dhj-10.1177_20552076231203893 for Using WhatsApp support groups to promote responsive caregiving, caregiver mental health and child development in the COVID-19 era: A randomised controlled trial of a fully digital parenting intervention by Sarah Skeen, Marguerite Marlow, Stefani du Toit, GJ Melendez-Torres, Lynette Mudekunye, Edwick Mapalala, Kelvin Ngoma, Byamukama Michael Ntanda, Moroesi Maketha, Caitlin Grieve, Laura Hartmann, Sarah Gordon and Mark Tomlinson in DIGITAL HEALTH

sj-pptx-2-dhj-10.1177_20552076231203893 - Supplemental material for Using WhatsApp support groups to promote responsive caregiving, caregiver mental health and child development in the COVID-19 era: A randomised controlled trial of a fully digital parenting intervention Click here for additional data file. Supplemental material, sj-pptx-2-dhj-10.1177_20552076231203893 for Using WhatsApp support groups to promote responsive caregiving, caregiver mental health and child development in the COVID-19 era: A randomised controlled trial of a fully digital parenting intervention by Sarah Skeen, Marguerite Marlow, Stefani du Toit, GJ Melendez-Torres, Lynette Mudekunye, Edwick Mapalala, Kelvin Ngoma, Byamukama Michael Ntanda, Moroesi Maketha, Caitlin Grieve, Laura Hartmann, Sarah Gordon and Mark Tomlinson in DIGITAL HEALTH

## Abbreviations

SS: Sarah Skeen
MaM: Marguerite Marlow
SdT: Stefani du Toit
GJMT: G.J. Melendez-Torres
LM: Lynette Mudekunye
EM: Edwick Mapalala
KN: Kelvin Ngoma
BMN: Byamukama Michael Ntanda
MoM: Moroesi Maketha
CG: Caitlin Grieve
LH: Laura Hartmann
SG: Sarah Gordon
MT: Mark Tomlinson
