# Supplementary figures and images for: Using WhatsApp support groups to promote responsive caregiving, caregiver mental health and child development in the COVID-19 era: A randomised controlled trial of a fully digital parenting intervention
Source: Digit Health. 2023 Nov 3;9:20552076231203893. doi: 10.1177/20552076231203893 (PMC10624105; doi:10.1177/20552076231203893)

## Slide 1
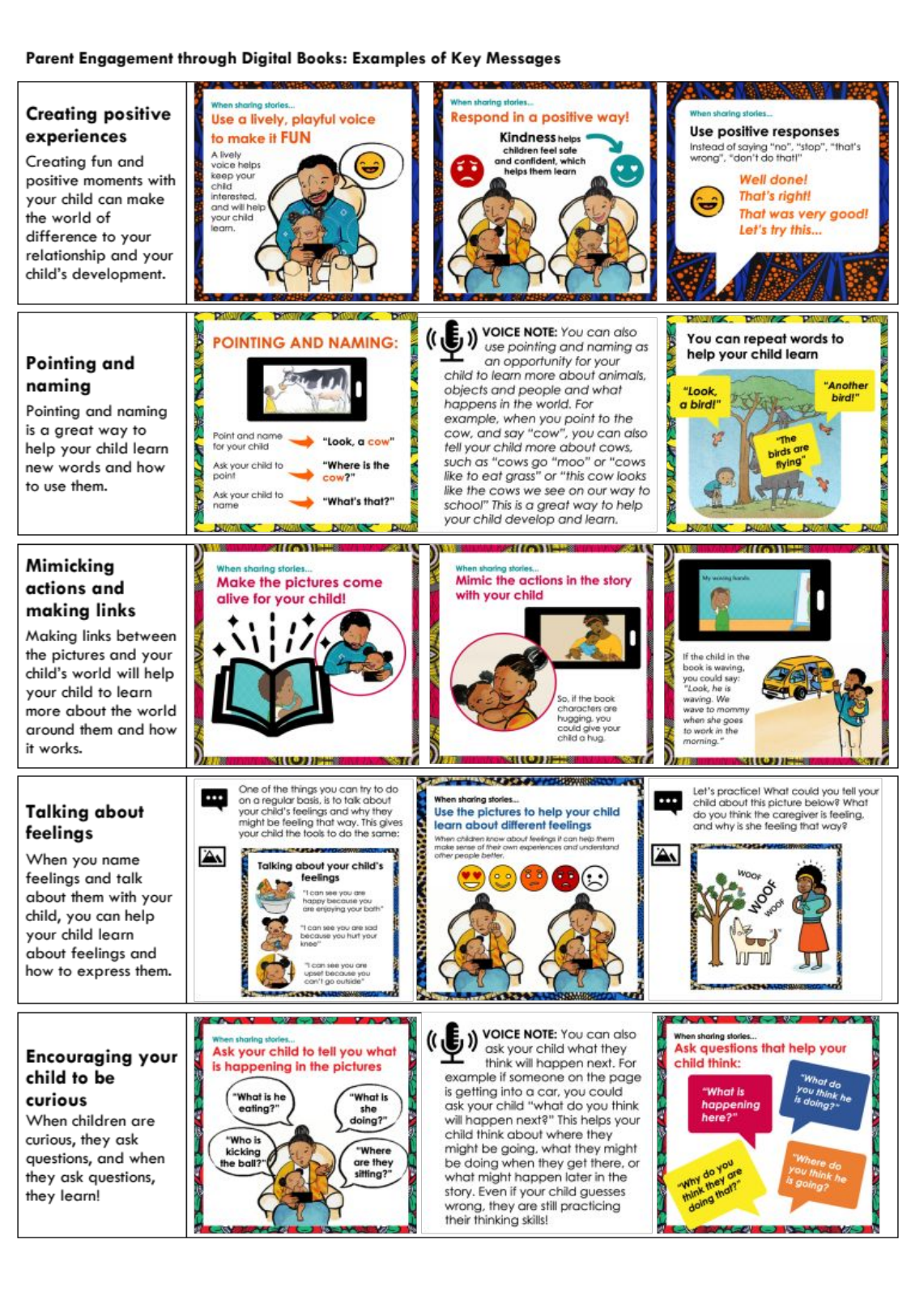

## Slide 2
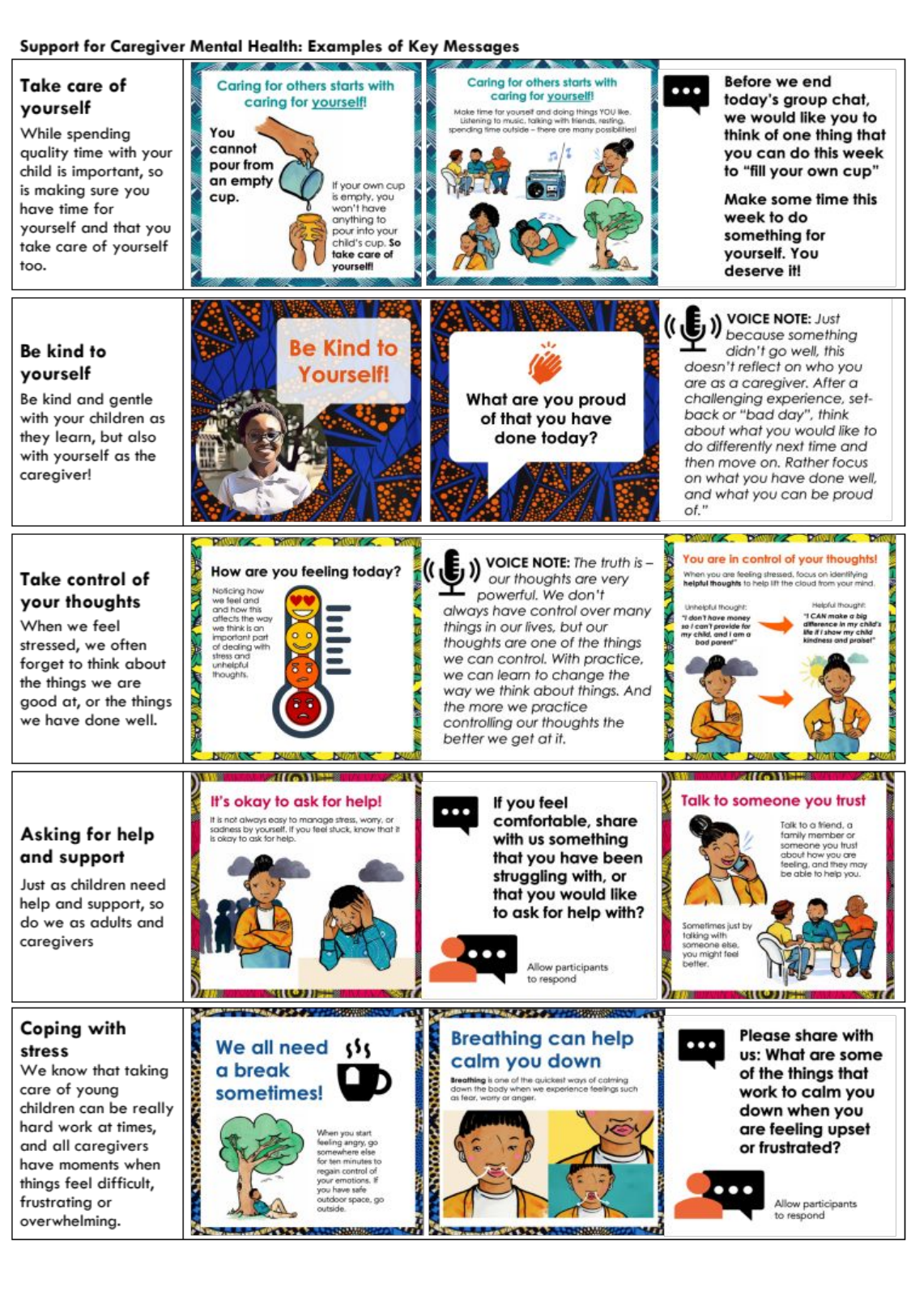

## Slide 3
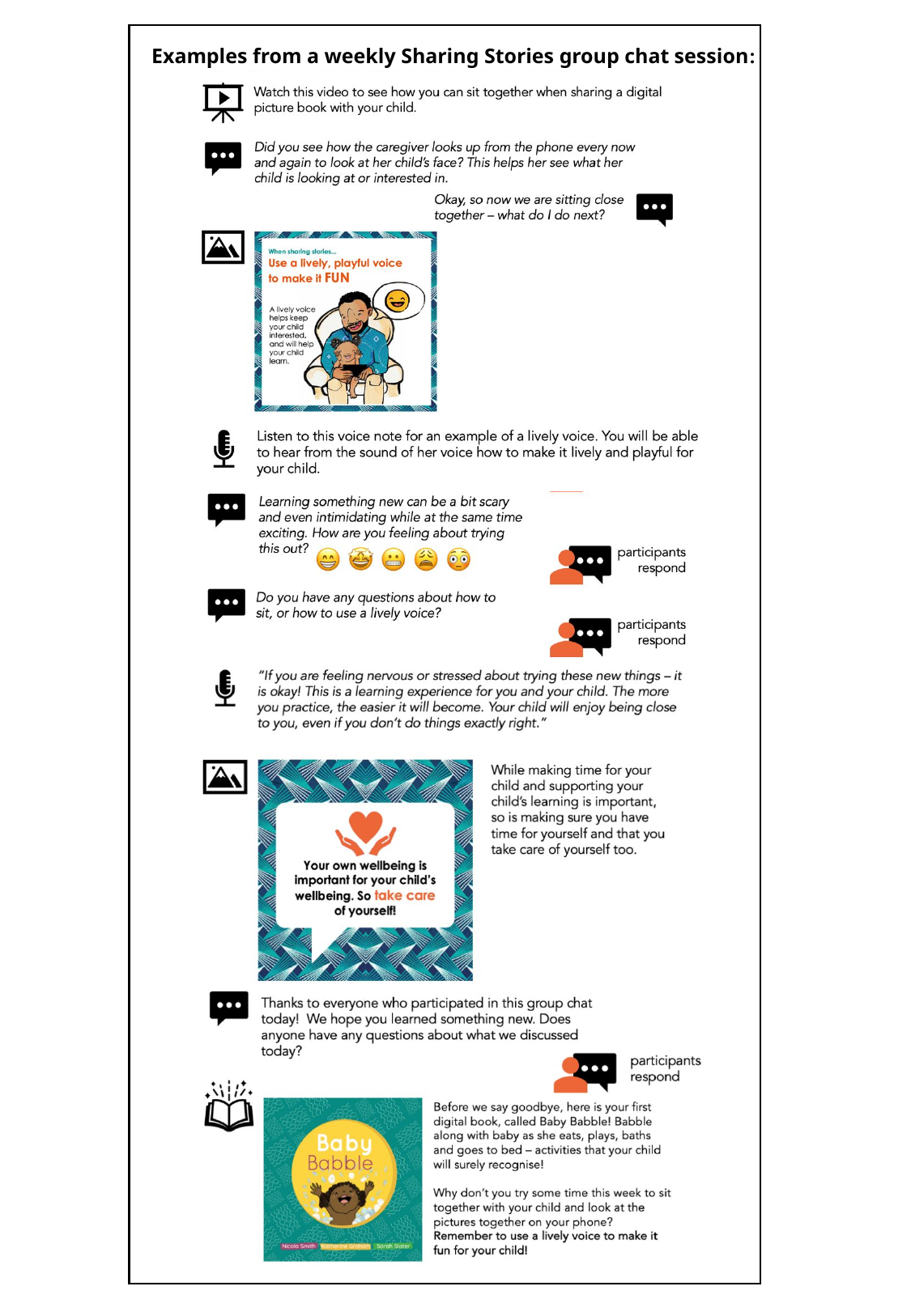

Examples from a weekly Sharing Stories group chat session:

Supplement: sj-pptx-2-dhj-10.1177_20552076231203893 - Supplemental material for Using WhatsApp support groups to promote responsive caregiving, caregiver mental health and child development in the COVID-19 era: A randomised controlled trial of a fully digital parenting intervention [file sj-pptx-2-dhj-10.1177_20552076231203893.pptx]
